# Genome wide scan for quantitative trait loci affecting tick resistance in cattle (*Bos taurus* × *Bos indicus*)

**DOI:** 10.1186/1471-2164-11-280

**Published:** 2010-04-30

**Authors:** Marco Antonio Machado, Ana Luisa S Azevedo, Roberto L Teodoro, Maria A Pires, Maria Gabriela CD Peixoto, Célio de Freitas, Márcia Cristina A Prata, John Furlong, Marcos Vinicius GB da Silva, Simone EF Guimarães, Luciana CA Regitano, Luiz L Coutinho, Gustavo Gasparin, Rui S Verneque

**Affiliations:** 1 Embrapa Gado de Leite, Juiz de Fora, MG, Brazil; 2 Departamento de Zootecnia, Universidade Federal de Viçosa, Viçosa, MG, Brazil; 3 Embrapa Pecuária Sudeste, São Carlos, SP, Brazil; 4 Escola Superior de Agricultura Luiz de Queiroz, Universidade de São Paulo, Piracicaba, SP, Brazil; 5 Departamento de Genética e Evolução, Universidade Federal de São Carlos, Rodovia Washington Luiz km 235, São Carlos/SP 13565-905, Brazil

## Abstract

****Background**:**

In tropical countries, losses caused by bovine tick *Rhipicephalus (Boophilus) microplus* infestation have a tremendous economic impact on cattle production systems. Genetic variation between *Bos taurus* and *Bos indicus* to tick resistance and molecular biology tools might allow for the identification of molecular markers linked to resistance traits that could be used as an auxiliary tool in selection programs. The objective of this work was to identify QTL associated with tick resistance/susceptibility in a bovine F2 population derived from the Gyr (*Bos indicus*) × Holstein (*Bos taurus*) cross.

**Results:**

Through a whole genome scan with microsatellite markers, we were able to map six genomic regions associated with bovine tick resistance. For most QTL, we have found that depending on the tick evaluation season (dry and rainy) different sets of genes could be involved in the resistance mechanism. We identified dry season specific QTL on BTA 2 and 10, rainy season specific QTL on BTA 5, 11 and 27. We also found a highly significant genome wide QTL for both dry and rainy seasons in the central region of BTA 23.

**Conclusions:**

The experimental F2 population derived from Gyr × Holstein cross successfully allowed the identification of six highly significant QTL associated with tick resistance in cattle. QTL located on BTA 23 might be related with the bovine histocompatibility complex. Further investigation of these QTL will help to isolate candidate genes involved with tick resistance in cattle.

## Background

In tropical regions, the incidence of the bovine tick *Rhipicephalus* (*Boophilus) microplus* deeply affects cattle production systems leading to a decrease in production and reproduction traits and even death of highly susceptible animals. Ticks, as blood-feeding parasites, affect their hosts both directly and as vector of viral, bacterial and protozoal diseases. Furlong *et al*. [1] found that 1/2 Gyr: 1/2 Holstein cows showed 23% decrease on milk production when the parasite load was ca. 105 ticks/cow.

Brazil has the largest commercial cattle herd in the world with more than 180 million animals and wastes approximately 390 million kg of meat/year (US$ 600 million) and 4 billion liters of milk/year (US$ 700 million) due to tick burdens [2]. In addition, infestation with ticks causes major losses in leather quality. Majority of Brazil's milk production is derived from crossbreds herds (Gyr × Holstein) also called Girolando. The importance of Gyr breed is paramount since it brings rusticity against heat and parasites to the highly productive Holstein breed in tropical regions [3].

Most of tick control is routinely accomplished by acaricides, however long term treatment has generated resistant strains. Use of acaricides, besides bringing additional costs to farmers, leaves chemical residues in meat, milk and in the environment. Vaccines have been used in some countries without solving the problem completely [4,5]. According de la Fuente *et al*. [6] a combination of commercial and technical problems has contributed to lack of vaccine usage.

*Bos indicus* breeds are known to be more resistant to ticks than *Bos taurus* breeds [3]. Since domestication took place in harsh environment, it could have guided the development of naturally resistant breeds. In this way, one alternative against tick burdens could be the use of resistant animals that could be correctly identified in the production systems with the aid of molecular tools.

Complex traits, such as disease resistance are under control of many genes, each with a different contribution on the phenotype. A Quantitative Trait Loci (QTL) is defined as a chromosomal segment showing a Mendelian transmission pattern with an effect on the trait of interest [7]. The identification of DNA markers linked to tick resistance would provide a better strategy for selecting resistant animals and could lead to the isolation of the genes underlying the resistance to ticks. Marker assisted selection (MAS) could be used to pre-select young animals, shorten generation interval and increase genetic gain [8,9]. Molecular markers have the capacity to allow prediction of breeding value for traits that had previously been difficult to measure and hence were not included in the selection criterion [8]. Understanding the biological and physiological mechanisms of these resistance genes could help to develop new and more effective acaricides and vaccines. In this study, we report the results of the first whole genome scan that led to the successful identification of six major QTL for tick resistance in the bovine genome.

## Methods

### Population

The experimental F2 population was produced by crossing four Holstein bulls with 27 Gyr cows to generate 150 F1 (1/2 Gyr : 1/2 Holstein) animals, using multiple ovulation with embryo transfer (MOET). Four F1 bulls were mated to 68 F1 females to generate 376 F2 animals, avoiding relationship among sires and cows. All F2 animals were raised together in the Embrapa Dairy Cattle experimental station, located in the southeast of Brazil. The weather can be divided in two seasons: mild and dry, from April to September, hot and humid, from October to March [10]. This population was produced from year 2000 to 2006.

The experimental calves were artificially reared on approximately 4 L of whole milk/day in individual houses up to 8 weeks of age in a tick free area. From this age up to six months, they were kept in paddocks of *Cynodon dactylon* L, supplemented with protein ration and chopped elephant grass (*Pennisetum purpureum*, Schumach). In these paddocks, they started to have contact with ticks. Thereafter, they were kept on pastures of *Brachiaria decumbens* (Stapf.) supplemented with chopped elephant grass and concentrate in the dry season. Minerals were made available in the paddocks for all animals. During the period from birth until 10 to 14 months of age, the F2 animals were not treated with any product to control ticks.

### Phenotype evaluation

To evaluate tick resistance, artificial infestations were performed in each F2 animal in the dry and rainy seasons. In this way, each animal was evaluated twice. The phenotypes for tick resistance were determined from 2001 to 2007 for 376 F2 animals based on evaluations in both seasons, distributed in 19 age groups. Tick evaluations were performed in these two different seasons to check the existence of distinct response mechanisms to tick infestation. The rainy season ranges from October to March; shows elevated temperatures (max 30°C and min 18°C) and average monthly precipitation of 147 mm. The dry season ranges from April to September, shows mild temperatures (max 26°C and min 13°C) and average monthly precipitation of 15 mm. It can be noted that the temperature between the two seasons are not so different but the monthly precipitation is nearly 10 times higher in the rainy season.

Infestation order was determined by the formation of each experimental group. This means that some animals had their first infestation in the dry season while some animals had their first infestation in the rainy season. As mentioned in the population topic, the first artificial infestation was not the first contact with ticks since this parasite is normally present on pasture in the experimental station.

Animals were evaluated in contemporary groups with age ranging from 10 to 14 months. Artificial infestation was carried out with approximately 10 000 *Rhipicephalus (Boophilus) microplus* larvae placed in the dorsal region of each animal. After that, animals were kept tied up for 30 min to avoid the self-grooming and to allow the larvae to spread to all regions of the body. After that, they were kept on pastures for 21 days when the engorged female ticks were counted [11].

Additional traits that might interfere with tick resistance were also evaluated, such as coat color, coat thickness, coat length and hair density. Coat color was determined by visual score according with the following classification: 1) totally white; 2) mostly white; 3) mostly dark; and 4) totally dark. Coat thickness was determined using a 0.05 mm precision pachimeter in three different regions of the animal body. This procedure evaluated the height of the lay down hair. Coat length was determined measuring the full length of the hair attached to the skin. Hair density was determined by removing hair samples from a determined area and counting the number of hair present in that area. These samples were removed from the same spots that coat thickness and coat length were evaluated.

### Genotyping

Blood samples from the parental, F1 and F2 generations were collected using vacuum tubes containing anti-clotting reagents. Genomic DNA was extracted from leukocytes using a modified phenol/chloroform method described shortly herein. Leukocytes were separated from fresh whole blood and transferred to 2 mL tubes. Samples were washed with lyses buffer until a white pellet was obtained. Pellet was treated with saline-proteinase K buffer and protein was removed by phenol-chloroform treatment. Quality and concentration of DNA were determined with the GeneQuant Pro spectrophotometer (GE Healthcare, Buckinghamshire, United Kingdom).

A total of 180 microsatellite markers were selected to cover the autosomal chromosomes with an average marker interval of 15 cM. Markers were selected from the map available at MARC/USDA (Meat Animal Research Center/United States Department of Agriculture) website: http://www.marc.usda.gov/genome/cattle/cattle.html. This map contains 3 802 microsatellite markers spanning 3 160 cM and has an average marker density of 1.4 cM [12-14]. Markers were chosen based on their location in the map, multi-allelism and minimum of 50% heterozygosity.

PCR reaction mixture contained 45 ng of genomic DNA, 0.8 U of Taq DNA polymerase, 0.2 mM of each dNTP, 0.1 μM of each primer, 1.5 or 2.0 mM of MgCl_2_, 20 mM Tris-HCl pH 8.3 and 50 mM KCl, in a final volume of 10 μL. Cycling parameters consisted of 35 cycles of 94°C for 30", annealing temperature (AT) for 30", 72°C for 30" and a final extension step at 72°C for 45'. Forward primers were 5' end labeled with fluorescent markers (6-FAM, HEX or TAMRA). The amplification reactions were performed with the GeneAmp PCR-System 9700 (Applied Biosystems, Foster City, CA, USA) and Mastercycler (Eppendorf, Hamburg, Germany).

Microsatellite marker alleles were detected by capillary electrophoresis in the MegaBACE 1000 DNA sequencer (GE Healthcare, Buckinghamshire, United Kingdom) or in the ABI Prism 3100 Avant sequencer (Applied Biosystems, Foster City, CA, USA).

Primer combinations were multiplexed based on the allelic range and fluorescent dyes before electrophoresis. Allele genotypes were determined with Fragment Profiler software (GE Healthcare, Buckinghamshire, United Kingdom) and data were exported to an Excel datasheet (Microsoft Corporation, Redmond, Washington) for markers analyzed in the MegaBACE 1000 DNA sequencer. For markers analyzed in the ABI Prism 3100 Avant sequencer, genotypes were determined using GENESCAN 3.7 and GENOTYPER 3.7.1 software (Applied Biosystems, Foster City, CA, USA).

### **Linkage Map**

Genetic linkage maps were generated using CriMap software [15] available at: http://linkage.rockefeller.edu/soft/crimap/. Recombination units were converted to cM using Kosambi's mapping function.

### Statistical analysis

The information content was calculated using the method described by Knott *et al*. [16]. Allelic frequencies, expected heterozygosity (He) and polymorphic information content (PIC) were calculated for the population using Cervus 2.0 software [17].

Tick count data did not follow normal distribution, so data was normalized using natural logarithmic transformation: log (tick count + 1) (Table 1). Hereafter the transformed data were called Log-tick. To determine if phenotypic data from rainy and dry seasons could be used as repetitive evaluations, the correlation between counts made in the same individual in two seasons was estimated using Pearson Product-Moment correlation coefficient.

**Table 1:** Distribution of tick count data before and after logarithmical transformation.

Tick count	Records	Log (tick count + 1)	Records
0-100	614	0-2	130
101-200	48	2-4	391
> 200	17	> 4	158

Analysis of variance (ANOVA) for tick resistance was performed using the PROC GLM function of SAS software (SAS Institute, Cary, NC), employing the general model: y = Xb + e, where *y* is the dependent variable (Log-tick), *X* is the incidence matrix of the fixed effects (sex, infestation order, coat color and year/group) and covariates (age at counting, hair density, coat length and coat thickness), and "e" is the random error ~ (0, σ_e_^2^). The significant sources of variation obtained from this analysis were used in the QTL analysis.

### QTL detection

QTL were mapped by regression analysis [18] using the F2 data analysis option available in the GridQTL software http://www.gridqtl.org.uk[19]. QTL alleles were assumed to be fixed or highly skewed in frequency between the breeds [20]. One-QTL model was computed in every cM along the chromosome.

F statistics was calculated to test the hypothesis of QTL segregation using a restricted model including year/group as fixed effect and coat thickness as covariate for both dry and rainy seasons. In addition, coat color was included as fixed effect in dry season only. Additive and additive + dominant effects were considered to detect QTL associated with tick resistance. The following models were used:

Additive:

Additive + dominant:

where,

Y_ijk_ = phenotype [log (tick count +1)]

μ = constant

G_i_ = year/group effect;

C_j_= coat color effect j: j = 1, 2, 3, 4; (only used in the dry season)

b = is the regression coefficient of Y on T,

T = coat thickness

e_ijk_ = error

c_a_ and c_d_ are functions of conditional probabilities of QTL given the marker genotypes, calculated as follows:

where:

P(QQ) = is the probability of homozygosis for the QTL genotypes derived from Holstein grandparents given the marker (M_i_M_i_; M_j_M_j_);

P(qq) = is the probability of homozygosis for the QTL genotypes derived from Gyr grandparents given the marker (M_i_M_i_; M_j_M_j_);

P(Qq) = is the probability of heterozygosis for the QTL genotypes given the marker (M_i_M_i_; M_j_M_j_);

The significance thresholds for 95% and 99% chromosome-wide significance were computed based on 10 000 permutations [21] and the confidence interval was estimated using the chi-square drop approximation [22].

The proportion of phenotypic variance was calculated using the formula [100 × (residual SS under Ho - residual SS under Ha)/(residual SS under Ho)] [23].

The P-value for a genome-wide (GW) significance level was obtained using the Bonferroni correction [24].

where *r* is the proportion of total genome length attributed to the chromosome.

## Results and Discussion

### Marker genotypes

The whole genome scan with 180 microsatellite markers generated 1 149 alleles with an average of 6.38 alleles/marker. All markers showed a segregation ratio of 1:2:1 with no segregation distortion. Marker CYP21 showed the highest number of alleles (15) and marker DIK4966 showed the lowest number of alleles (2) (Additional File 1). For most cases, the number of alleles found in the F2 population was smaller than the number of alleles reported on MARC map (Additional File 1). This high allelic variation found on MARC map was somehow expected since it was generated from many breeds of cattle [13-15]. A small number of founder animals (31) from two cattle breeds (Gyr and Holstein) were used to generate the F2 population and, because of that, a smaller number of alleles were found in this population in comparison to MARC data.

The total number of alleles and the allele frequency (data not shown) were different among populations - Holstein, Gyr, F1 and F2. The average number of alleles found in the Holstein population was 3.2 alleles/marker while the Gyr population showed a higher diversity of 5.9 alleles/marker. This discrepancy in the allele number between these two populations might be due to different number of sampled animals in each population (four Holstein and 27 Gyr). For the F1 and F2 populations, the average number of alleles was 6.6 alleles/marker. It was found a great variation for the allele frequencies between the parental populations indicating a great diversity between Holstein and Gyr breeds [25].

In F2 populations, QTL mapping is based on the genetic variation among founder lineages and requires that the parental populations carry fixed alternate alleles at the QTL [26]. Nevertheless, F2 populations are not generally formed by 100% contrasting lineages or breeds. This fact was well noticed in this study since 25% of the detected alleles were shared between the two parental breeds Gyr and Holstein. A total of 643 marker alleles (55.9%) were found only in the Gyr breed and 216 alleles were found only in the Holstein breed.

For the F2 population, the average value of PIC was 0.67. Marker CYP21 showed the highest PIC value (0.87) and marker BMC1013 showed the lowest PIC value (0.24) (Additional File 1). Suggested PIC value classes are: PIC > 0.5 (high polymorphism); 0.25 < PIC < 0.5 (moderate polymorphism) and PIC < 0.25 (low polymorphism) [27]. According to these criteria, the great majority of markers genotyped in the F2 population might be considered highly polymorphic. PIC values are the most common indexes to determine the extent of the polymorphism of a marker [28,29] and the usefulness of a marker in segregation analysis is directly related to its level of polymorphism. Despite of the small number of Gyr founder animals, it is possible to select markers with high PIC values and high number of alleles to set a panel for paternity tests and genealogy studies in this breed. Markers BM4440, IOBT959, NLBCMK13, CYP21 and DIK5183 fit these criteria and could be selected for that.

All chromosome linkage maps generated in this study with the F2 population agreed in marker order with the Marc/USDA bovine linkage maps. BTA 1, 2, 3, 6, 9, 11, 13, 16, 17, 18, 20, 22, 23, and 27 showed approximately the same length as the MARC map. BTA 4, 5, 7, 8, 10, 12, 14, 21, 24, 25, 26, 28, and 29 were found to be larger compared to MARC map. BTA 15 and 10 were the only linkage maps found to be shorter than MARC maps (Additional File 1). This was somehow expected since the F2 population generated a smaller number of recombinant meioses compared to MARC map.

### **Tick resistance phenotype**

The average number of ticks did not differ between the two seasons, with average values of 44 ± 59 in the dry season and 41 ± 69 in the rainy season. In natural infestations, it is normally found a seasonal effect on tick number per animal with higher numbers found in the dry season [30,31]. These different results may be explained by the great influence of environmental factors in the tick free life cycle that affect the number of larvae. In this work, the animals were artificially inoculated with 10 000 larvae each. The absence of difference between the average tick counts in the two seasons may reflect a higher effect of environment factors in larvae population and viability in the pasture than the larvae survival in the host contributing to differences in natural infestations.

The estimated heritability for tick resistance in the F2 population was 0.21 ± 0.12 Log-tick indicating genetic variation for this trait. The average heritability for tick resistance from several studies reviewed by Davis [32] was 0.34.

The correlation between Log-tick in the two seasons was low (0.36) indicating that the evaluations in each season are distinct phenotypes. Considering this result, we did not include consecutive tick counts as repetitive evaluations and all analyses were performed separately for rainy and dry season. Although the average number of ticks in the two seasons was the same, the individual animal response was different for each season. Once our results suggest that the environmental conditions did not affect the parasite life cycle it is possible to consider that these individual variation were related to seasonal variations in the immunological response of the F2 animals against tick challenge.

The analysis of variance (Table 2) indicated that year/group, coat color and coat thickness affected the Log-tick in the dry season while only year/group and coat thickness affected the Log-tick in the rainy season. Animals with whiter coat color showed less ticks than animals with dark coat color (Table 3). These results agree with other works indicating that white coat color animals showed less ticks in relation to dark coat color animals [31,33]. According to these authors, the higher susceptibility to tick of dark coat color animals could be probably caused by a decrease in the level of resistance since these animals might be more affected by the heat stress in the rainy season. Some properties of the hair coat and coat color in cattle enhance conductive and convective heat loss and reduce absorption of solar radiation [33]. Another explanation would be that in whiter animals ticks are more visible to predators such as birds if compared to animals with dark coat color [2].

**Table 2:** Analysis of variance for the Log-tick in the Embrapa F2 population.

Effect	DF^#^	Rainy season		Dry season	
		
		F value	P	F value	P
Sex	1	0.10	ns	0.02	ns
infest	1	1.89	ns	3.25	ns
year/group	14	14.60	< 0.0001	6.40	< 0.0001
Age	1	0.29	ns	1.13	ns
coat color	3	1.35	ns	5.04	0.002
coat thickness	1	6.18	0.014	5.09	0.025
Hair density	1	0.39	ns	0.64	ns
coat length	1	2.39	ns	0.86	ns

^#^ Degrees of freedom

ns - non-significant

**Table 3:** Least square means (LSM) and standard error (SE) for Log-tick for coat color in the Embrapa F2 population.

Coat color	Rainy season		Dry season	
	
	LSM	SE	LSM	SE
Totally white	2.63	0.302	2.47	0.261
Predominance white	2.70	0.168	2.86	0.143
Predominance dark	2.94	0.096	3.23	0.070
Totally dark	3.11	0.185	3.43	0.171

Coat thickness showed significant effect on the tick count in both rainy and dry seasons (Table 2). It has been reported that animals with short hair are more resistant to ticks than animals with long hair [34]. According to these authors, a thicker coat might favor the parasite since it creates a microclimate that helps keeping the parasite attached to the surface of the animal. In addition, thicker coat complicates the animal's self-grooming that helps removing attached ticks.

It was somehow expected that the response of the animals would be different between the first and second artificial infestation, but this has not been verified. According to Bonsma & Pretorius [35], bovine tick resistance can be innate or acquired. The level of resistance increases after various infestations compared to the resistance level at the first infestation [36,37]. This was not found in this study since the infestation order was non-significant in the ANOVA. One likely reason for this result would be that the F2 animals had already been in contact with ticks naturally present in pastures before being artificially infested. Thus, the first artificial infestation was not the first contact of the animal with the parasite.

### QTL mapping

**Figure 1:**
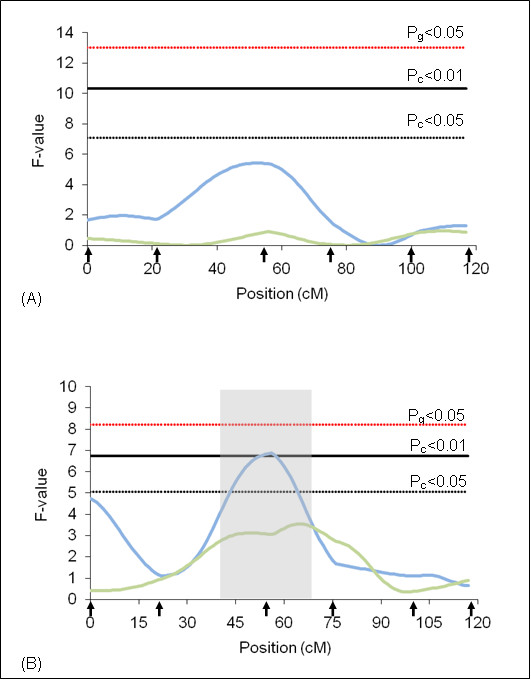
**F-statistic profile for tick resistance on BTA2**. The x-axis indicates the relative position in the linkage map. Arrows indicate marker positions. Green line indicates rainy season and blue line indicates dry season. Gray bar indicates QTL confidence interval. P_g_ = genome wide significance threshold and P_c_ = chromosome wide significance threshold. (A) analyses results using additive model and (B) analyses results using additive + dominant models.

The interval analysis was able to identify QTL regions for tick resistance with distinct results in the dry and rainy seasons. We identified six QTL, three in the rainy season, two in the dry season and one in both seasons. Three QTL were identified with chromosome-wide significance P_c_ < 0.01 on BTA 2, BTA10 and BTA27 and three QTL were identified with genome-wide significance P_g_ < 0.05 on BTA5, BTA11 and BTA23. Summarized results of F-value, QTL location, confidence interval and QTL effects from significant QTL found are shown in Table 4. Graphic results of the detected QTL are shown in the Figures 1 through 6 in detail.

**Table 4:** Summary of the genome wide scan in the Embrapa F2 population.

BTA	Season	F-value		Position	0,95	Start	End	Adjusted	Additive	Dominance	%σ^2^
		A	AD	cM	CI	CI	CI	Mean	effect	Effect	
2	Dry	5.4	6.9**	56	22	43	65	1.76	-0.19	-0.32	4.22

5	Rainy	16.3^#^	8.2**	140	20	129	149	2.82	-0.39	0.05	5.57

10	Dry	12.2**	6.4*	19 and	47	32	79	1.54	0.37	0.18	4.00

11	Dry	7.2*	4.0					1.52	0.24	0.14	

	Rainy	16.5^##^	8.6**	43	26	27	53	2.46	0.39	0.12	5.26

23	Dry	19.7^##^	9.9^#^	22	12	18	30	1.73	0.38	-0.07	5.90

	Rainy	18.9^##^	9.7^#^	32	17	25	42	2.79	0.40	-0.11	5.82

27	Rainy	11.1**	5.5*	0	12	0	12	2.71	0.32	0.01	3.31

A - Additive model

AD - Additive + Dominance model

CI - Confidence interval (cM)

σ^2^ - Proportion of phenotypic variance explained by the QTL

* P_c_ < 0.05 - Chromosome-wide

** P_c_ < 0.01 - Chromosome-wide

^#^ P_g_ < 0.05 - Genome-wide

^##^ P_g_ < 0.01 - Genome-wide

On BTA2, a QTL (Pc < 0.01) for the dry season was detected in the middle of the chromosome (Figure 1) under the additive plus dominance model. The additive effect was not significant indicating that this QTL has dominant effect, which according to the model used, implies that heterozygous animals show a lower number of ticks when compared to the average of homozygous individuals. This QTL explains 4.22% of the total phenotypic variation for the trait (Table 4). The average Log-tick for this chromosome was 1.8 ± 0.3 and the dominant effect associated to the QTL causes a change in the average of -0.3.

**Figure 2:**
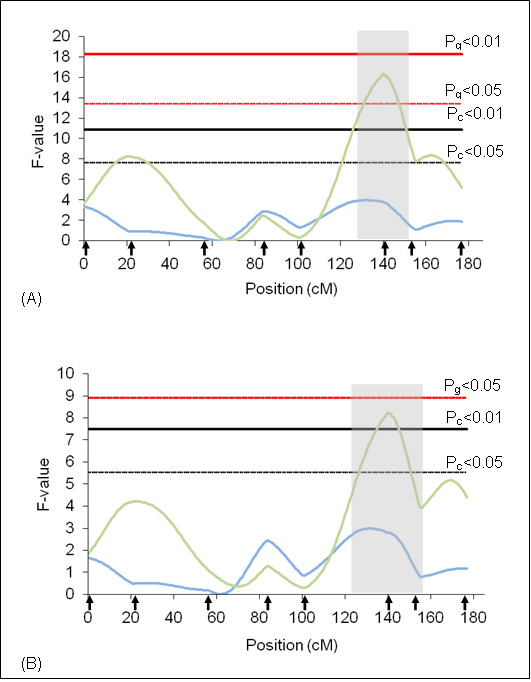
**F-statistic profile for tick resistance on BTA5**. The x-axis indicates the relative position in the linkage map. Arrows indicate marker positions. Green line indicates rainy season and blue line indicates dry season. Gray bar indicates QTL confidence interval. P_g_ = genome wide significance threshold and P_c_ = chromosome wide significance threshold. (A) analyses results using additive model and (B) analyses results using additive + dominant models.

On BTA5, a QTL (Pg < 0.05) for the rainy season was detected near the telomeric end of the chromosome (Figure 2), using the additive model. This QTL explains 5.50% of the total phenotypic variation for the trait (Table 4). The additive effect is negative, indicating that alleles originated from Holstein cause a decrease in the number of ticks. This was the only one case among the QTL described here were Holstein alleles decreased the average tick count. Finding these Holstein derived resistance alleles was somewhat unexpected since this breed is less resistant to ticks compared to Gyr breed. This QTL would require a deeper investigation in order to understand the inheritance of resistance attributed to this region.

**Figure 3:**
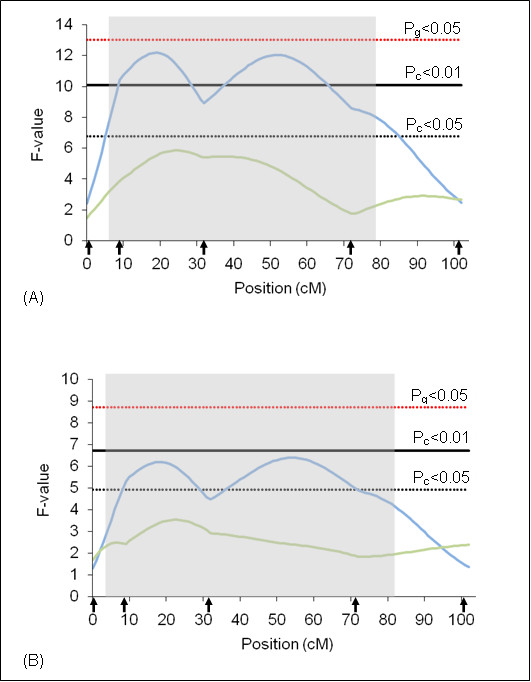
**F-statistic profile for tick resistance on BTA10**. The x-axis indicates the relative position in the linkage map. Arrows indicate marker positions. Green line indicates rainy season and blue line indicates dry season. Gray bar indicates QTL confidence interval. P_g_ = genome wide significance threshold and P_c_ = chromosome wide significance threshold. (A) analyses results using additive model and (B) analyses results using additive + dominant models.

On BTA10, two QTL peaks (P_c_ < 0.01) for the dry season were detected under the additive model (Figure 3). This QTL explains 4.00% of the total phenotypic variation for the trait (Table 4). The additive effect was positive, indicating that alleles originated from Holstein contributed to increase in the number of ticks/animal. These two peaks suggest that two QTL might be explaining the variation for the trait. New markers should be added in this region to decrease the confidence interval and determine the number of QTL involved on BTA 10.

**Figure 4:**
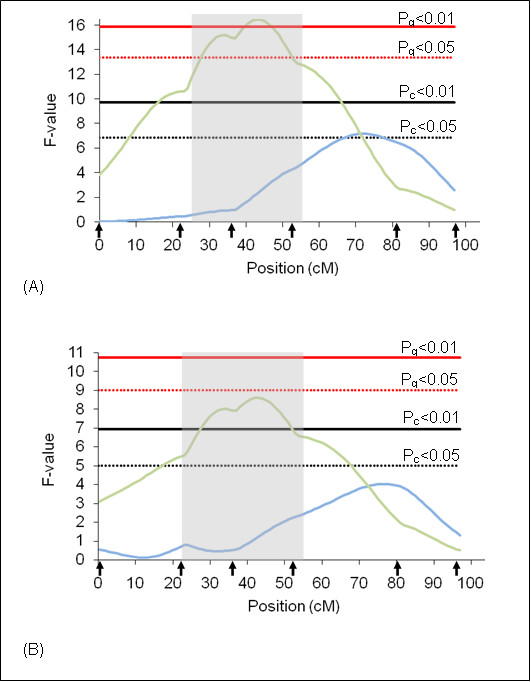
**F-statistic profile for tick resistance on BTA11**. The x-axis indicates the relative position in the linkage map. Arrows indicate marker positions. Green line indicates rainy season and blue line indicates dry season. Gray bar indicates QTL confidence interval. P_g_ = genome wide significance threshold and P_c_ = chromosome wide significance threshold. (A) analyses results using additive model and (B) analyses results using additive + dominant models.

On BTA11, a QTL (P_g_ < 0.01) for the rainy season was detected in the central part of the chromosome (Figure 4), using the additive model. This QTL explains 5.26% of the total phenotypic variation for the trait (Table 4). The average Log-tick for this chromosome was 2.5 ± 0.3 and additive effect causes a change in the average of 0.41.

**Figure 5:**
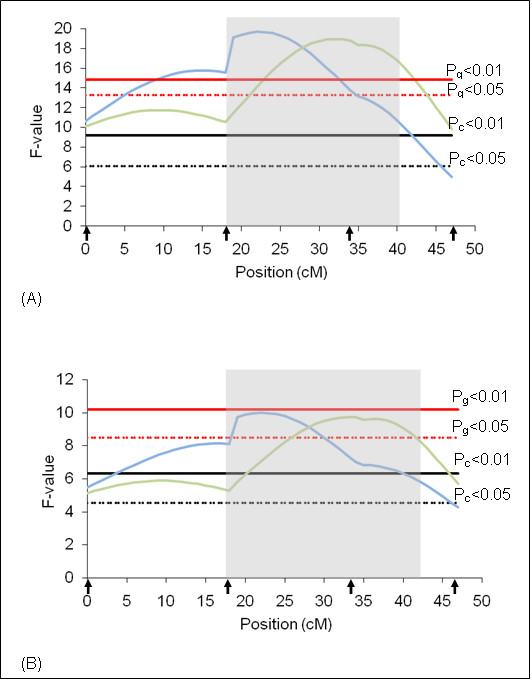
**F-statistic profile for tick resistance on BTA23**. The x-axis indicates the relative position in the linkage map. Arrows indicate marker positions. Green line indicates rainy season and blue line indicates dry season. Gray bar indicates QTL confidence interval. P_g_ = genome wide significance threshold and P_c_ = chromosome wide significance threshold. (A) analyses results using additive model and (B) analyses results using additive + dominant models.

BTA 23 was the only chromosome in which significant QTL (P_g_ < 0.01) were found on both rainy and dry seasons (Figure 5). The phenotypic variation explained by this QTL was 5.9% for the dry season and 5.7% for the rainy season. The additive effect was positive indicating that tick resistance was originated from Gyr alleles. In this same chromosome, it was detected an interaction of BoLA DRB3 gene with tick resistance in this same F2 population [2]. BoLA DRB3 gene belongs to bovine histocompatibility complex and is directly related to inflammatory processes. These loci encode for surface molecules relevant in the induction and regulation of the immune response [38]. Acosta-Rodriguez *et al*. [39] showed that some MHC BoLA class II alleles determine, at least partly, the susceptibility to tick infestation. BoLA class I alleles w6.1 and w7 have been related to tick protection. Cattle with antigens w6.1 and w7 had significantly fewer ticks than cattle lacking these antigens [40-42]. The BoLA complex is located on BTA23q2.1 and maps within the confidence interval of this QTL indicating that a gene or genes of this complex might be underlying this QTL. Fine mapping of this region with additional markers should help to deeper understand this possible interaction.

**Figure 6:**
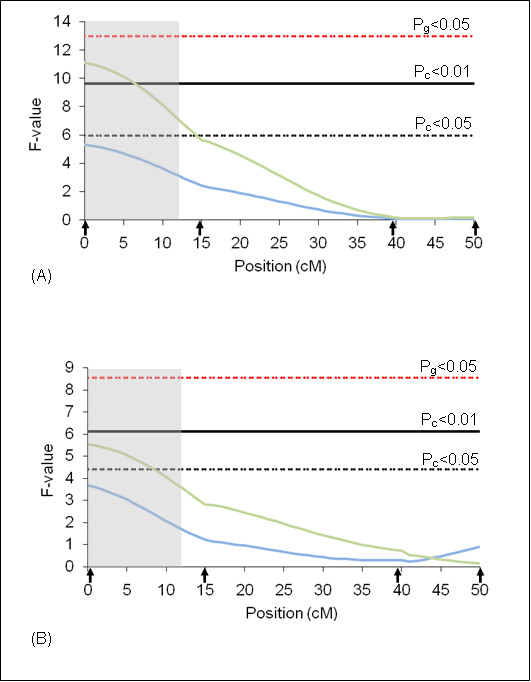
**F-statistic profile for tick resistance on BTA27**. The x-axis indicates the relative position in the linkage map. Arrows indicate marker positions. Green line indicates rainy season and blue line indicates dry season. Gray bar indicates QTL confidence interval. P_g_ = genome wide significance threshold and P_c_ = chromosome wide significance threshold. (A) analyses results using additive model and (B) analyses results using additive + dominant models.

On BTA27, a QTL (P_c_ < 0.01) for the rainy season was detected in the beginning part of the chromosome (Figure 6), using additive model. This QTL explains 3.31% of the total phenotypic variation for the trait (Table 4). Apparently, the distribution of F values along the chromosome for both wet and dry seasons show the same pattern although significant value was detected only in the rainy season.

Gasparin *et al*. [43] genotyped selected chromosomes and detected tick resistance QTL on BTA5, 7 and 14 using this same Embrapa F2 population. These results were based on preliminary phenotypic data of 300 F2 animals and included putative QTL with higher threshold (p < 0.1). Regitano *et al*. [44] reported additional ongoing results using this same population and detected QTL on BTA 4, 5, 7, 10, 11, 14, 18 and 23 with chromosome-wide threshold ranging from 0.01 < p < 0.1. After publication of these partial results, additional 60 animals were phenotyped and the remaining chromosomes were genotyped in order to cover the whole bovine genome. Additional miss paternities that arouse from the new marker genotype information were discarded. The association studies were re-processed and we were able to calculate the genome-wide threshold for each chromosome. We detected various putative QTL (p < 0.05) and some of them were the ones previously reported by Gasparin *et al*. [43] and Regitano *et al*. [44] (data not shown). In this current work, we only report highly significant QTL detected below 1% chromosome-wide threshold to minimize the chance of detecting false-positive QTL.

The use of QTL information detected in a line cross is less directly applicable to a pure breed population compared to a QTL detected in the same breed of interest. Nevertheless, the QTL results detected in our F2 population (Gyr × Holstein) could be more easily transferred to commercial herds of dairy cattle in Brazil, since the vast majority of these herds are constituted by Gyr × Holstein crosses.

Fine mapping with additional markers in regions where significant QTL were detected for tick resistance could increase the level of significance for QTL as well as decrease the confidence interval. This would greatly increase the applicability of markers assisted selection by lowering the probability of occurrence of crossing over between the marker and QTL. Analysis of data from different breeds or populations might also provide additional insights into the genes controlling the trait of interest. In addition, the refinement of QTL position will allow a more precise location of the QTL limits, thus aiding in the identification of orthologous genes [45] into segments of human or mice, which can provide a list of candidate genes that may explain the effect of QTL for tick resistance.

Bovine resistance to ticks is a complex mechanism and a variety of physiological pathways might be directly involved such as general inflammatory reactions that could avoid or make difficult the fixation of tick in the animal. In this way, many immunology related genes described in other species may be related to bovine tick resistance.

The recent release of the bovine genomic sequencing project [46] and the HapMap project [47], which made available ultra high-density SNP chips, will facilitate the search for the causative mutations underlying the QTL and help understanding the physiological mechanisms involved in bovine tick resistance.

## **Conclusions**

We have successfully identified six QTL regions strongly associated with tick resistance in cattle on the chromosomes BTA 2, 5, 10, 11, 23 and 27. QTL located on BTA 23 might be related with the bovine histocompatibility complex. Further investigation of these QTL will help to isolate genes involved in tick resistance mechanisms in cattle.

## Authors' contributions

MAM was the leader of the project and did most of the writing of the final version of this manuscript. As part of her Ph.D. dissertation, ALSA carried out most of the microsatellite genotyping, data analysis and helped writing the manuscript. RSV and RLT participated in the project conception and contributed with the statistical analyses. MCAP, JF, MFAP and RLT were responsible for generating the phenotypic trait data. CF was in charge of the *in vitro* embryo production and animal husbandry. MGCDP and MVGBS were responsible for refining QTL mapping analyses. SEFG, LLC, LCAR and GG were responsible for genotyping selected chromosomes, helped on establishing lab procedures and QTL analysis routines. All authors read and approved the manuscript.

## Supplementary Material

Additional file 1 **Summary of genome wide scan markers used to genotype the Embrapa F2 population**. (Marker name); number of alleles from MARC/USDA map (MARC Alleles); number of alleles found on Embrapa F2 population (Embrapa F2 Alleles);	marker location on MARC/USDA map (MARC	 map); marker location on Embrapa map (Embrapa F2 map); Polymorphic Information Content detected on Embrapa F2 population (PIC Embrapa F2). Click here for file
