# Supplementary material for: Genome wide scan for quantitative trait loci affecting tick resistance in cattle (Bos taurus × Bos indicus)
Source: BMC Genomics. 2010 Apr 30;11:280. doi: 10.1186/1471-2164-11-280 (PMC2880304; doi:10.1186/1471-2164-11-280)
Supplement: Additional file 1 — Summary of genome wide scan markers used to genotype the Embrapa F2 population. (Marker name); number of alleles from MARC/USDA map (MARC Alleles); number of alleles found on Embrapa F2 population (Embrapa F2 Alleles); marker location on MARC/USDA map (MARC map); marker location on Embrapa map (Embrapa F2 map); Polymorphic Information Content detected on Embrapa F2 population (PIC Embrapa F2). [file 1471-2164-11-280-S1.DOC]

**Additional File 1** – Summary of genome wide scan markers used to genotype the Embrapa F2 population.

| **BTA** | **Marker** | **MARC*** | **Embrapa** | **MARC** | **Embrapa F2** | **PIC** |
| --- | --- | --- | --- | --- | --- | --- |
|  | **name** | **Alleles** | **F2 Alleles** | **map** | **map** | **Embrapa F2** |
| 1 | AGLA17 | 4 | 3 | 0 | 0 | 0.5 |
|  | BM8139 | 7 | 6 | 10 | 12 | 0.5 |
|  | BMS711 | 9 | 7 | 24 | 26 | 0.7 |
|  | BMS4024 | 9 | 10 | 45 | 44 | 0.7 |
|  | BMS4009 | 8 | 7 | 76 | 71 | 0.7 |
|  | BM864 | 14 | 6 | 100 | 95 | 0.7 |
|  | BMS1789 | 11 | 9 | 114 | 109 | 0.8 |
|  | BMS599 | 14 | 8 | 139 | 142 | 0.8 |
| 2 | TGLA44 | 16 | 8 | 4 | 0 | 0.8 |
|  | MNB-83 | 4 | 3 | 29 | 22 | 0.6 |
|  | BM4440 | 11 | 10 | 60 | 56 | 0.9 |
|  | TGLA226 | 12 | 7 | 86 | 76 | 0.8 |
|  | BMS2519 | 12 | 10 | 110 | 103 | 0.8 |
|  | IDVGA-2 | 12 | 8 | 126 | 118 | 0.8 |
| 3 | DIK4651 | 10 | 8 | 0 | 0 | 0.6 |
|  | INRA006 | 8 | 4 | 17 | 12 | 0.5 |
|  | BMS2904 | 6 | 4 | 27 | 19 | 0.3 |
|  | BL41 | 10 | 8 | 43 | 40 | 0.3 |
|  | BM220 | 6 | 7 | 66 | 66 | 0.8 |
|  | HUJII77 | 11 | 8 | 87 | 89 | 0.8 |
|  | DIK2904 | 9 | 6 | 116 | 114 | 0.6 |
| 4 | BL1024 | 7 | 5 | 8 | 0 | 0.7 |
|  | BMS1634 | 9 | 5 | 31 | 29 | 0.6 |
|  | MAF70 | 11 | 6 | 46 | 52 | 0.7 |
|  | INRA072 | 12 | 10 | 63 | 77 | 0.7 |
|  | BMS648 | 6 | 6 | 91 | 113 | 0.8 |
|  | TCRB | 2 | 3 | 101 | 145 | 0.4 |
| 5 | BM6026 | 10 | 6 | 6 | 0 | 0.7 |
|  | BP1 | 16 | 6 | 17 | 21 | 0.6 |
|  | BM321 | 7 | 3 | 38 | 56 | 0.4 |
|  | BMS1617 | 11 | 6 | 56 | 84 | 0.7 |
|  | BMS490 | 8 | 7 | 66 | 101 | 0.8 |
|  | BMS1248 | 19 | 7 | 91 | 140 | 0.6 |
|  | ILSTS034 | 12 | 10 | 103 | 155 | 0.8 |
|  | ETH152/ MB006 | 6 | 6 | 122 | 177 | 0.6 |
| 6 | ILSTS093 | 21 | 10 | 0 | 0 | 0.7 |
|  | DIK4498 | 8 | 5 | 20 | 19 | 0.7 |
|  | DIK1058 | 7 | 6 | 38 | 38 | 0.7 |
|  | MNB-208 | 7 | 9 | 60 | 60 | 0.7 |
|  | DIK4867 | 7 | 7 | 82 | 82 | 0.7 |
|  | DIK2995 | 5 | 4 | 110 | 113 | 0.6 |
|  | DIK2690 | 4 | 5 | 121 | 122 | 0.7 |
|  | JMP12 | 4 | 4 | 134 | 135 | 0.4 |
| 7 | INRA192 | 9 | 7 | 82 | 0 | 0.7 |
|  | BM9065 | 11 | 4 | 101 | 32 | 0.7 |
|  | ILSTS006 | 10 | 4 | 117 | 46 | 0.6 |
|  | BM7160 | 10 | 4 | 0 | 47 | 0.6 |
|  | BM2607 | 3 | 3 | 30 | 90 | 0.6 |
|  | BOBT24 | 7 | 5 | 32 | 119 | 0.7 |
|  | IL4 | 12 | 5 | 32 | 150 | 0.7 |
|  | BM6117 | 5 | 3 | 62 | 189 | 0.3 |
| 8 | Z27077 | 10 | 5 | 11 | 0 | 0.6 |
|  | BM310 | 6 | 6 | 31 | 36 | 0.5 |
|  | BM4006 | 9 | 7 | 50 | 69 | 0.6 |
|  | BMS2072 | 10 | 5 | 66 | 96 | 0.6 |
|  | BB703 | 12 | 9 | 77 | 107 | 0.8 |
|  | BM711 | 13 | 8 | 93 | 121 | 0.8 |
|  | CSSM047 | 8 | 5 | 119 | 149 | 0.5 |
| 9 | ETH225 | 10 | 7 | 13 | 0 | 0.8 |
|  | ILSTS037 | 10 | 7 | 26 | 13 | 0.8 |
|  | BMS817 | 9 | 7 | 42 | 34 | 0.7 |
|  | UWCA9 | 10 | 7 | 50 | 43 | 0.8 |
|  | BMS2377 | 7 | 3 | 71 | 65 | 0.6 |
|  | BMS2251 | 8 | 3 | 87 | 81 | 0.6 |
|  | BMS1943 | 9 | 9 | 104 | 102 | 0.8 |
| 10 | BM1237 | 9 | 9 | 25 | 0 | 0.8 |
|  | BL1035 | 11 | 8 | 32 | 9 | 0.8 |
|  | BM875 | 7 | 7 | 54 | 32 | 0.7 |
|  | INRA037 | 16 | 5 | 79 | 72 | 0.6 |
|  | BMS2614 | 10 | 8 | 109 | 103 | 0.8 |
| 11 | BMS424B | 9 | 8 | 15 | 0 | 0.8 |
|  | INRA177 | 9 | 6 | 35 | 24 | 0.7 |
|  | BM7169 | 17 | 7 | 50 | 37 | 0.7 |
|  | RM150 | 14 | 9 | 70 | 54 | 0.8 |
|  | BL1103 | 13 | 8 | 98 | 81 | 0.8 |
|  | BMS655 | 7 | 6 | 117 | 97 | 0.6 |
| 12 | DIK4746 | 7 | 8 | 3 | 0 | 0.7 |
|  | BMS2252 | 12 | 8 | 14 | 16 | 0.8 |
|  | IOBT959 | 9 | 12 | 46 | 66 | 0.8 |
|  | DIK4789 | 4 | 5 | 65 | 110 | 0.6 |
|  | DIK4227 | 6 | 6 | 88 | 129 | 0.6 |
|  | DIK4583 | 8 | 4 | 109 | 149 | 0.6 |
| 13 | TGLA23 | 11 | 4 | 9 | 0 | 0.4 |
|  | BMC1222 | 14 | 7 | 28 | 22 | 0.7 |
|  | UWCA25 | 9 | 7 | 59 | 49 | 0.8 |
|  | AGLA232 | 15 | 10 | 91 | 91 | 0.7 |
| 14 | CSSM066 | 10 | 7 | 5 | 0 | 0.8 |
|  | ILSTS011 | 6 | 3 | 26 | 17 | 0.6 |
|  | BMC1207 | 11 | 4 | 52 | 49 | 0.4 |
|  | BMS740 | 10 | 6 | 61 | 68 | 0.8 |
|  | BMS1899 | 10 | 6 | 69 | 90 | 0.7 |
|  | BL1036 | 10 | 8 | 100 | 116 | 0.8 |
|  | BMS2055 | 10 | 4 | 94 | 143 | 0.6 |
| 15 | DIK2777 | 13 | 11 | 0 | 0 | 0.8 |
|  | JAB8 | 9 | 7 | 31 | 30 | 0.8 |
|  | MS2245 | 4 | 4 | 41 | 37 | 0.6 |
|  | DIK2313 | 8 | 7 | 58 | 51 | 0.7 |
|  | DIK2768 | 10 | 9 | 78 | 65 | 0.8 |
|  | DIK5382 | 12 | 9 | 101 | 84 | 0.8 |
| 16 | MGTG1 | 5 | 3 | 0 | 0 | 0.6 |
|  | DIK4303 | 6 | 4 | 16 | 29 | 0.5 |
|  | DIK2654 | 5 | 5 | 30 | 64 | 0.7 |
|  | DIK4635 | 10 | 6 | 46 | 84 | 0.7 |
|  | DIK673 | -- | 5 | -- | 98 | 0.6 |
|  | DIK2296 | -- | 6 | -- | 116 | 0.7 |
|  | DIK4011 | -- | 7 | -- | 138 | 0.5 |
| 17 | DIK2332 | 10 | 6 | 0 | 0 | 0.4 |
|  | DIK2105 | 4 | 5 | 19 | 17 | 0.5 |
|  | DIK5322 | 7 | 9 | 34 | 31 | 0.8 |
|  | DIK5152 | 7 | 6 | 46 | 40 | 0.7 |
|  | DIK2910 | 5 | 8 | 64 | 61 | 0.8 |
|  | DIK4383 | 5 | 8 | 78 | 76 | 0.7 |
|  | MS2263 | 6 | 8 | 94 | 102 | 0.7 |
| 18 | BMS2559 | 7 | 8 | 1 | 0 | 0.6 |
|  | ILSTS021 | 6 | 4 | 11 | 8 | 0.6 |
|  | BMS2213 | 11 | 10 | 24 | 25 | 0.8 |
|  | BM8151 | 9 | 7 | 40 | 45 | 0.7 |
|  | BMS2639 | 13 | 8 | 56 | 58 | 0.8 |
|  | BB710 | 6 | 6 | 62 | 67 | 0.7 |
|  | TGLA227 | 14 | 7 | 84 | 89 | 0.7 |
| 19 | DIK2574 | 14 | 13 | 0 | 0 | 0.8 |
|  | HEL10 | 7 | 6 | 16 | 14 | 0.7 |
|  | BP20 | 9 | 8 | 46 | 40 | 0.8 |
|  | CSSM065 | 9 | 4 | 70 | 59 | 0.6 |
|  | IDVGA-44 | 11 | 3 | 86 | 74 | 0.6 |
|  | BMC1013 | 7 | 3 | 107 | 88 | 0.2 |
| 20 | NRDIKM004 | 4 | 8 | 1 | 0 | 0.6 |
|  | NRDIKM009 | 7 | 8 | 17 | 11 | 0.7 |
|  | NLBCMK13 | 10 | 12 | 30 | 21 | 0.8 |
|  | NRDIKM012 | 7 | 6 | 46 | 43 | 0.7 |
|  | NRDIKM002 | 7 | 6 | 60 | 52 | 0.7 |
|  | DIK553 | 5 | 9 | 83 | 74 | 0.7 |
| 21 | DIK4593 | 5 | 9 | 1 | 0 | 0.8 |
|  | DIK2272 | 5 | 3 | 14 | 13 | 0.5 |
|  | DIK4001 | 11 | 7 | 30 | 29 | 0.8 |
|  | MNB-88 | 6 | 9 | 45 | 48 | 0.7 |
|  | AFZ1 | 8 | 11 | 68 | 73 | 0.8 |
|  | DIK3023 | 8 | 5 | 84 | 106 | 0.5 |
| 22 | DIK1161 | 5 | 5 | 1 | 0 | 0.6 |
|  | MNS-20 | 7 | 8 | 22 | 21 | 0.8 |
|  | DIK2694 | 5 | 6 | 32 | 29 | 0.7 |
|  | CSSM058 | 5 | 5 | 45 | 40 | 0.6 |
|  | DIK2030 | 6 | 5 | 62 | 63 | 0.6 |
|  | UW49 | 8 | 6 | 76 | 79 | 0.7 |
|  | DIK5307 | 8 | 9 | 88 | 91 | 0.8 |
| 23 | BM1815 | 8 | 8 | 24 | 0 | 0.8 |
|  | CYP21 | 11 | 15 | 42 | 18 | 0.9 |
|  | BM1818 | 8 | 8 | 58 | 34 | 0.8 |
|  | BM1905 | 11 | 10 | 72 | 47 | 0.8 |
| 24 | MNB60 | -- | 12 | -- | 0 | 0.8 |
|  | DIK5395 | 5 | 7 | 16 | 31 | 0.6 |
|  | DIK4426 | 7 | 5 | 32 | 54 | 0.7 |
|  | MNB90 | -- | 8 | -- | 108 | 0.6 |
|  | DIK4971 | 7 | 9 | 78 | 137 | 0.8 |
| 25 | DIK5183 | 9 | 10 | 4 | 0 | 0.7 |
|  | TGLA40 | 7 | 8 | 16 | 16 | 0.8 |
|  | DIK2077 | 8 | 6 | 31 | 32 | 0.6 |
|  | DIK614 | 6 | 5 | 43 | 50 | 0.6 |
|  | BM1864 | 9 | 5 | 68 | 84 | 0.6 |
| 26 | ABS012 | 6 | 6 | 3 | 0 | 0.7 |
|  | DIK4513 | 6 | 5 | 24 | 36 | 0.5 |
|  | DIK1055 | 8 | 6 | 32 | 67 | 0.8 |
|  | DIK727 | 4 | 5 | 50 | 90 | 0.5 |
|  | DIK2279 | 7 | 7 | 62 | 111 | 0.7 |
|  | DIK2724 | 6 | 5 | 79 | 131 | 0.7 |
| 27 | BM3507 | 17 | 10 | 0 | 0 | 0.7 |
|  | BMS2137 | 6 | 6 | 21 | 15 | 0.7 |
|  | CSSM036 | 9 | 7 | 43 | 40 | 0.8 |
|  | INRA027 | 6 | 6 | 58 | 51 | 0.7 |
| 28 | DIK4742 | 4 | 4 | 0 | 0 | 0.4 |
|  | DIK5300 | 10 | 8 | 15 | 27 | 0.7 |
|  | DIK2448 | 8 | 10 | 29 | 45 | 0.8 |
|  | DIK713 | 12 | 7 | 46 | 60 | 0.8 |
|  | DIK1143 | 6 | 6 | 62 | 78 | 0.7 |
| 29 | INRA143 | 7 | 5 | 0 | 0 | 0.7 |
|  | DIK4188 | 5 | 3 | 16 | 26 | 0.4 |
|  | BMS1600 | 6 | 5 | 29 | 59 | 0.6 |
|  | DIK4966 | 2 | 2 | 32 | 82 | 0.3 |
|  | RM040 | 5 | 4 | 40 | 101 | 0.5 |
|  | DIK5057 | 5 | 4 | 70 | 137 | 0.6 |
